# Editorial

**Published:** 1847-05

**Authors:** 


### THE MEDICAL EXAMINER.

PHILADELPHIA, MAY, 1847.

### INHALATION OF SULPHURIC ETHER.

Our Exchange journals continue to be crowded with cases and discussions illustrative of the effects of this kind of medication. Not merely is it employed when cutting instruments are used, but in the treatment of luxations, hernia, and labour, especially in instrumental cases; in asthma, croup, and nearly all the ills that flesh is heir to. These numerous experiments will doubtless lead to a better understanding of its powers and adaptations, and it will be strange if much good does not result. Bad consequences are known to have followed the employment of the remedy in repeated instances, but the only wonder is, with such indiscriminate use of so powerful an agent, that the number of accidents has not been greater. That our readers may know something of what is said and doing on the subject, at home and abroad, we have, at the sacrifice of variety, devoted a considerable portion of the present number to extracts bearing on the most important points involved in the discussion. Already, in this country, we think we discover a decline of the excitement in relation to it, which prevailed so extensively at the first.

### DELEGATES TO THE NATIONAL MEDICAL CONVENTION.

For the following list of Delegates to the National Medical Convention, we are indebted chiefly to the American Journal of the Medical Sciences. We have made some additions to the list published in that journal, but still the roll is not complete; and of those named it is not likely that all will attend, while the places of some will probably be supplied by others. It is understood that representatives have been appointed in the States of Connecticut and Louisiana, but we are not advised of their names or how many have been designated.

Vermont.—Drs. Chas. Hall, C. W. Horton, A. G. Dana, and Dyer Story.

Massachusetts.—From the *Massachusetts Medical Society*, Drs. S. W. Williams, of Deerfield, E. Hale, of Boston, E. Huntingdon, of

Lowell, A. L. Pierson, of Salem, R. Fowler, of Stockbridge, J. V. C. Smith, of Boston, L. Bartlett, of New Bedford, E. W. Carpenter, of Sandwich, W. Bridgeman, of Springfield, O. W. Holmes, of Boston, and Geo. C. Shattuck, jr., of Boston.

From *Middlesex District Medical Society*, Drs. Elisha Huntingdon, John W. Graves, Nehemiah Cutter, and Josiah Curtis.

Rhode Island.—From the *Rhode Island Medical Society*, Drs. Theophilus C. Dunn, Usher Parsons, Richmond Brownell, and George Carpenter.

New York.—From *New York State Medical Society*, Drs. John Stearns, J. W. Francis, J. C. Cheeseman, J. R. Manley, E. G. Ludlow, J. A. Wing, Danl. Ayers, T. W. Blachford, Darius Clark, Morgan Snyder, Jas. S. Sprague, J. M'Call, A. Willard, N. S. Davis, P. H. Hard, Maltby Strong, Alex. Thompson, L. T. Tefft, G. W. Bradford, Enos Barnes.

From *New York Academy of Medicine*, Drs. J. Stearns, F. C. Stewart, J. R. Wood, D. H. Bulkley, V. Mott, E. Delafield, J. C. Bliss, R. S. Kissam, D. M. Reese, E. L. Beadle, J. Linsley, O. S. Bartles, C. S. Smith, M. Hoit, W. H. Van Buren, J. O. Pond.

From the *New York Medical and Surgical Society*, Drs. J. A. Swett, J. G. Adams, A. Dubois, A. C. Post, W. P. Buel.

From the *New York Hospital*, Drs. John Watson, and John H. Griscom.

From *Albany Medical College*, Profs. Marsh, J. M'Naughton, T. R. Beck, and Hun.

From Faculty of *Geneva Medical College*, Profs. C. A. Lee, C. B. Coventry, and J. Webster.

From *Faculty of Medicine of Buffalo University*, Prof. F. H. Hamilton, Austin Flint, J. P. White, and Geo. Hadley.

New Jersey.—From *Medical Society of New Jersey*, Drs. Smith, and Pierson, of Essex, C. Marsh, of Passaic, Stewart, of Morris, Forman, of Mercer, Parrish, of Burlington, Taylor and Cooper, of Camden, Garrison, of Gloucester, and Howell.

From *District Medical Society of Burlington*, Drs. Cole, Stratton, and Read.

Pennsylvania.—From *College of Physicians of Philadelphia*, Drs. T. T. Hewson, I. Hays, S. Jackson, J. W. Moore, A. Stillé, J. R. Paul, W. Pepper, G. Fox, C. Morris, D. F. Condie, J. Randolph, R. C. Bridges, C. D. Meigs.

From *University of Pennsylvania*, Profs. N. Chapman, S. Jackson, Geo. B. Wood.

From *Jefferson Medical College*, Profs. Bache, Mitchell, and Mütter.

From *Med. Dep. of Pennsylvania College*, Profs. Atlee, Patterson, and Wiltbank.

From *Franklin Medical College*, Profs. Rogers, Tucker, and Joynes.

From *Philadelphia Medical College*, Profs. Burden and M'Clintock.

From *Philadelphia Medical Society*, Drs. Bell, Emerson, Parrish, Norris, West, Ashmead, B. H. Coates, Bond, Morton, Yardley, Griscom, and Rodman.

From *Northern Medical Association of Philadelphia*, Drs. Janney, Naudain, Uhler, Remington, M. B. Smith, and Jewell.

From *Lancaster Co. Medical Society*, Drs. Humes, Atlee, Kerfoot, Eshleman, Winters, sen., Duffield and Carpenter.

VIRGINIA.—*Medical Convention of Virginia*, Drs. Welford, of Fredericksburg, Cabell, of the University, W. A. Patterson, of Richmond, and M'Guire, of Winchester.

OHIO.—*Ohio Medical Convention*, Prof. Wright, of Med. Coll. of Ohio, Prof. St. John, of Med. Coll. of Cleveland, and Prof. Butterfield, of Med. Coll. of Willoughby.

GEORGIA.—*Medical College of Georgia*, Profs. Dugas and Garvin.

MISSISSIPPI.—*Mississippi State Medical Society*, Drs. S. A. Cartwright, H. Lipscombe, —— Monette, P. D. Ewing, J. S. Copes, Wm. R. Gist, O. L. Dewees, K. P. Alston, G. G. Banks, W. J. Leake, J. Andrews, Geo. Nicholson, G. Keïrn, T. J. Catchings, W. Jamison, E. D. Turner, —— Takett, J. R. Sykes, —— Kilpatrick, Wm. Balfour, —— Davis, James Maynard, James Chamberlain, and S. C. Farrar.

KENTUCKY.—*University of Louisville*, Profs. Drake, Cobb, and Yandell.

From *Transylvania University*, Professors Mitchell and Bartlett.

MISSOURI.—*Medical Department of University of Missouri*, Prof. J. B. Johnston.

*Medical Department of St. Louis University*, Prof H. M. Bullit.

TENNESSEE.—*Medical Society of Tennessee*, Drs. J. B. Hays, Stout, Martin, Buchanan, Avent, and Davidson.

DELAWARE.—Drs. Couper, and Thompson.

——

*Arrangements for the Meeting of the National Medical Convention.*

At a meeting of the Delegates to the National Medical Convention from the city and county of Philadelphia, held at the Hall of the College of Physicians, March 9th, 1847, it was resolved to accept the polite offer made by the *Academy of Natural Sciences*, of the use of their spacious Hall for the meeting of the Convention; and the following committee was appointed to make the necessary arrangements for the meetings and deliberations of that body:—Drs. Hays, Condie, Emerson, Fox, Bridges, Norris, Morris, West, and Paul.

The above committee, in furtherance of the objects of their appointment, invite the delegates to the National Medical Convention to meet at the Hall of the Academy of Natural Sciences, west side of Broad Street, near Chestnut Street, on Wednesday, May 5th, at 10 o'clock A. M.

The several standing committees appointed at the last Convention are invited to meet at the same place on Monday morning, May 3d, at 10 o'clock.

To facilitate intercourse between the delegates, they are invited to report themselves as soon after their arrival in Philadelphia as convenient, to the committee of reception and arrangement, named above, who will be at the Hall of the Academy of Natural Sciences on the 1st, 3d, and 4th of May, from 10 A. M. to 3 P. M., and on the evening of the 4th of May from 7 to 10 o'clock.

The secretaries of the associations who will be represented are requested to transmit, at an early day, the names of their delegates to the chairman of the committee, Dr. I. Hays.

